# Encapsulation of Nanocrystals in Mannitol-Based Inhalable Microparticles via Spray-Drying: A Promising Strategy for Lung Delivery of Curcumin

**DOI:** 10.3390/ph17121708

**Published:** 2024-12-18

**Authors:** Luca Casula, Emanuela Fabiola Craparo, Eleonora Lai, Cinzia Scialabba, Donatella Valenti, Michele Schlich, Chiara Sinico, Gennara Cavallaro, Francesco Lai

**Affiliations:** 1 Department of Life and Environmental Sciences, University of Cagliari, S.P. Monserrato-Sestu km 0.700, Monserrato, 09042 Cagliari, Italy; luca.casula@unica.it (L.C.); eleonora.lai4@unica.it (E.L.); donatella.valenti@unica.it (D.V.); michele.schlich@unica.it (M.S.); sinico@unica.it (C.S.); 2 Laboratory of Biocompatible Polymers, Department of Biological, Chemical and Pharmaceutical Sciences and Technologies (STEBICEF), University of Palermo, Via Archirafi 32, 90123 Palermo, Italy; emanuela.craparo@unipa.it (E.F.C.); cinzia.scialabba@unipa.it (C.S.); gennara.cavallaro@unipa.it (G.C.)

**Keywords:** nano into micro, nanosuspension, inhalable microparticles, curcumin, pulmonary delivery

## Abstract

Background/Objectives: Curcumin is well known for its great anti-inflammatory and antioxidant efficacy, representing a potential strategy for the treatment of respiratory disorders. However, several drawbacks, such as chemical instability, poor water solubility and rapid metabolism, result in low bioavailability, limiting its clinical applications. In this study, curcumin nanocrystals were incorporated into mannitol-based microparticles to obtain an inhalable dry powder. Methods: A curcumin nanosuspension was produced by wet-ball media milling and thoroughly characterized. Spray drying was then used to produce mannitol microparticles incorporating curcumin nanocrystals. In vitro release/dissolution tests were carried out in simulated lung fluids, and the aerosolization properties were evaluated using a Next-Generation Impactor (NGI, Apparatus E Ph. Eu.). Results: The incorporation of curcumin nanocrystals into mannitol-based microparticles influenced their morphological properties, such as geometric diameters, and flowability. Despite these changes, nebulization studies confirmed optimal MMAD values (<5 µm), while multi-step dissolution/release studies evidenced the influence of mannitol. Conclusions: The developed curcumin nanocrystals-loaded mannitol microparticles show promise as an inhalable treatment for respiratory diseases, combining effective aerodynamic properties with controlled drug release.

## 1. Introduction

Lung inflammation is the primary cause of several deadly pulmonary conditions, such as lung cancer, pulmonary fibrosis, COPD, pneumonia, asthma and pneumocystis. Numerous types of inflammatory cells are activated to restore lung homeostasis, playing a delicate balance between inflammation and anti-inflammation mediators. However, persistent pro-inflammatory states can result in the onset of chronic illnesses [1,2]. Plant-based medications can be used as a possible remedy for respiratory conditions, as they contain active natural molecules—like flavonoids, alkaloids, and terpenoids—that can exert various therapeutic activities, such as anti-inflammatory, antiviral, antiplatelet, antitumor, antiallergic and antioxidant activities [3,4]. Among them, the natural polyphenol curcumin (CUR), which comes from the plant Curcuma longa, has attracted a lot of interest because of its ability to modulate cytokines, enzymes and transcription factors implicated in a large number of medical conditions and diseases [5]. In the context of lung health, CUR has shown promise in reducing inflammation and oxidative stress, two major contributors to diseases like lung cancer, asthma and chronic obstructive pulmonary disease (COPD), demonstrating its therapeutic potential [6,7,8,9]. However, an unstable chemical structure, limited water solubility, rapid metabolism and poor absorption and bioavailability are some of CUR’s disadvantages [10]. The scientific community has been working extensively to address the challenges related to poorly soluble substances in drug delivery, especially using nanotechnology. To this aim, various nanocarriers—such as liposomes, cyclodextrins, cubosomes and polymeric nanoparticles—can be used to encapsulate hydrophobic active substances or drugs [11,12,13,14]. The production of nanoparticles of pure drugs without any matrix materials, formerly known as drug nanocrystals, represents another effective strategy [15,16,17]. They are usually prepared as an aqueous nanosuspension using either bottom-up or top-down methods, and they are stabilized by polymers or surfactants [18,19]. Nanocrystals can also be embedded into redispersible microparticles to obtain inhalable dry powders, combining the favorable features of both the nano- and micro-systems [20,21]. In fact, microparticles are known to exhibit favorable aerodynamic properties and quickly dissolve in contact with mucus on the respiratory surface, releasing the nanocrystals, whose particles size and surface properties can considerably improve the drug dissolution and absorption in the lungs [22,23]. Among the various microparticles engineering techniques, spray-drying—a single-step manufacturing process—is frequently used to convert the nanosuspension into dry powders [24]. However, during the strong desiccation process of spray-drying, nanocrystals can adhere to each other, resulting in particle aggregation, an alteration of the crystal structure and changes in the interfacial properties [25]. To overcome this issue, several sugars (e.g., lactose, trehalose) and sugar alcohols (e.g., mannitol, xylitol) have been identified as optimal matrix agents that improve powder reconstitution performance by preventing nanocrystals aggregation [26]. In our study, a CUR nanosuspension was produced through wet ball media milling and subsequently incorporated into mannitol-based microparticles via spray-drying, using different drug-to-mannitol weight ratios. The characterization of CUR-nanosuspension included the determination of the zeta potential size by dynamic and electrophoretic light scattering (DLS; ELS) and morphological analysis by SEM, while that of the microparticles included the evaluation of their morphology and flowability and the determination of aerosolization parameters. The physical–chemical and technological properties of the obtained systems demonstrated the potential of the produced formulations for inhalation therapy.

## 2. Results and Discussion

### 2.1. Preparation and Characterization of CUR-Nanosuspension

**Figure 1:**
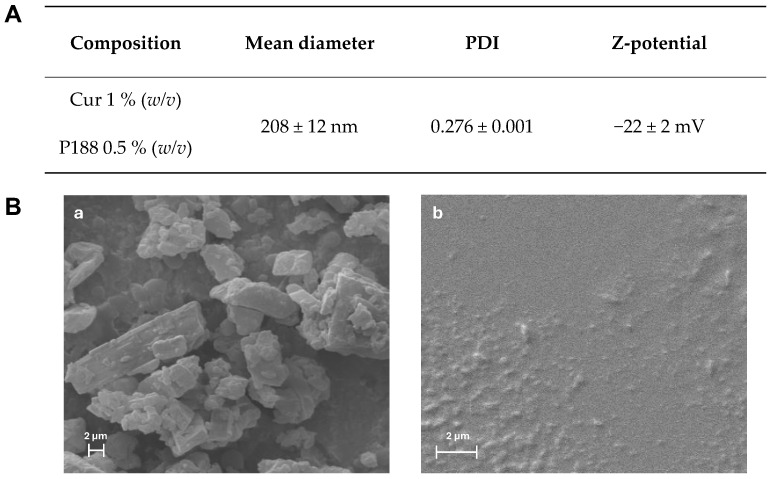
(**A**) Composition and DLS data of the CUR-nanosuspension and (**B**) SEM micrographs of (**a**) curcumin raw powder and (**b**) CUR-nanosuspension.

In this work, the wet ball media milling technique was used to obtain CUR nanocrystals as nanosuspension (CUR-nanosuspension), using precise ratios of the selected ingredients. A 1% CUR-nanosuspension formulation with a small average diameter and PDI was designed in a prior study by optimizing the milling time (70 min) and the concentration of Poloxamer 188 [27], a non-ionic linear low toxic copolymer used in many commercially available products [28]. Figure 1A reports the prepared CUR-nanosuspension composition and chemical–physical characteristics such as the mean diameter, PDI and Z-potential.

CUR-nanosuspension presented a moderately narrow size distribution (Supplementary Figure S1), with a mean diameter of 208 nm and a polydispersity index (PDI) of approximately 0.28. Interestingly, it was reported that CUR nanocrystals stabilized with P188, having a particle size < 500 nm, showed improved mucus diffusion and lung epithelium uptake [29]. Using the M3-PALS technique, the zeta potential value was found to be relatively weakly negative (−22 mV). Nonetheless, the nanosuspension formulation’s long-term stability was demonstrated in our prior study [27]. Environmental scanning electron microscopy has been used to examine the morphology of raw CUR powder and CUR-nanosuspension. As the raw CUR powder shows large and irregularly elongated crystals (Figure 1B-a), the CUR-nanosuspension shows a uniform particle size distribution and a regular, rounded shape of nanocrystals (Figure 1B-b), highlighting the effect of the high shear forces involved in the milling process on the size reduction [30]. Even though there seemed to be a slight tendency for the nanocrystals to aggregate, this might be related to the sample preparation for SEM analysis. As a matter of fact, prior to gold sputtering, a drop of formulation was applied to the stub and allowed to dry. The resulting evaporation of water may cause an agglomeration of the nanocrystals, which is only an operative artifact, since the PDI value derived from the DLS analysis demonstrates a narrow size distribution [31].

### 2.2. Preparation and Characterization of Microparticles

One common bottom-up processing technique for creating engineered particles for inhalation is spray drying (SD). By appropriately modifying the process variables and the properties of the feed fluid, i.e., the qualitative–quantitative composition, the SD technique enables the modulation and optimization of the powder macroscopic characteristics (flowability, bulk and tapped density, etc.) as well as the particle physical–chemical and technological properties (size, density, morphology, surface area, porosity, etc.) [32,33]. Here, SD was used to produce inhalable microparticles. Because of its remarkable stability during samples production, its pharmaceutical safety and its ability to rehydrate the airways, mannitol was selected as the main component of the microparticles. Through its osmotic effect, it is able to alter the mucus viscoelastic properties, enhancing mucociliary clearance [34,35]. Through SD, the CUR-nanosuspension was converted into a solid product, in which the solid nanocrystals (from here on called CUR-NC) are dispersed into the mannitol matrix of microparticles.

**Figure 2:**
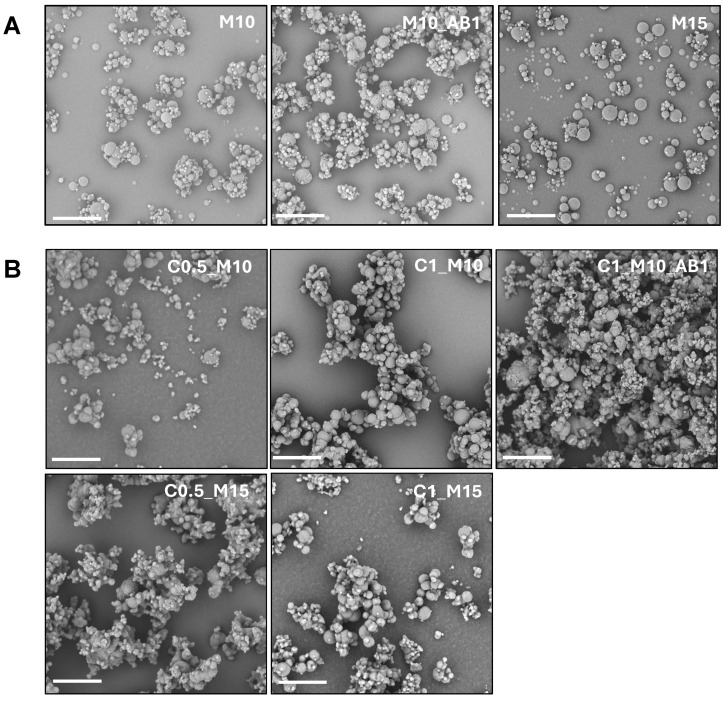
SEM images of (**A**) the produced empty microparticle samples M10, M10_AB1 and M15, and (**B**) the produced curcumin-loaded microparticle samples C0.5_M10, C1_M10, C1_M10_AB1, C0.5_M15 and C1_M15. (magnification 3000×; the bar represents 20 µm).

Several microparticle samples were produced in order to investigate the effect of the concentration of various components on the final properties of the obtained microparticles. In particular, the matrices were obtained from an aqueous feed suspension by varying the concentration of CUR (0%, 0.5% or 1% *w*/*v*) and the concentration of mannitol (10% or 15% *w*/*v*), obtaining, respectively, samples M10, M15, C0.5_M10, C0.5_M15, C1_M10 and C1_M15. Samples M10_AB1 and C1_M10_AB1 were obtained, respectively, by adding ammonium bicarbonate (AB) to the liquid feed containing CUR 1% *w/v* and mannitol 10% *w*/*v*. AB was added because it is well known in the literature as an optimal porogen agent when used to produce inhalable particles via SD, as it forms pores after decomposition and the release of volatile products. Here, the use of AB is justified by the intent to verify whether its addition would result in the formation of particles with better aerodynamic characteristics compared to matrices obtained without AB [36,37]. The SEM images of the samples obtained in the absence and presence of CUR-NC are shown in Figure 2A,B, respectively, while the yield, DL and EE values are presented in Table 1.

As observed from the images, the presence of CUR-NC seems to promote the microparticle aggregation. However, the Hausner Index (reported also in Table 1) indicates that its addition improves the flowability compared to the empty microparticles, particularly in samples with a lower CUR concentration (samples C0.5_M10 and C0.5_M15). The addition of AB leads to a deterioration of the flow properties of the sample C1_M10 (1.76 vs. 1.57) and a significant reduction in the powder yield.

The geometric diameter (d_geom_), calculated on an adequate number of particles, as described in the experimental section, decreases in almost all the samples in the presence of CUR_NC, notwithstanding the polydispersity of the samples’ increases. Notably, the sample obtained in the presence of AB and CUR shows an extensive presence of aggregates, as already observed in the SEM images and in the change of the flow properties, resulting in an impossibility of measuring the d_geom_.

A crucial step in characterizing pharmaceutical products is the evaluation of any structural alterations that might occur during production procedures or as a result of incompatibilities with the excipients used [38]. To obtain preliminary information on this point, DSC was used to analyze the single components and the final formulations.

**Figure 3:**
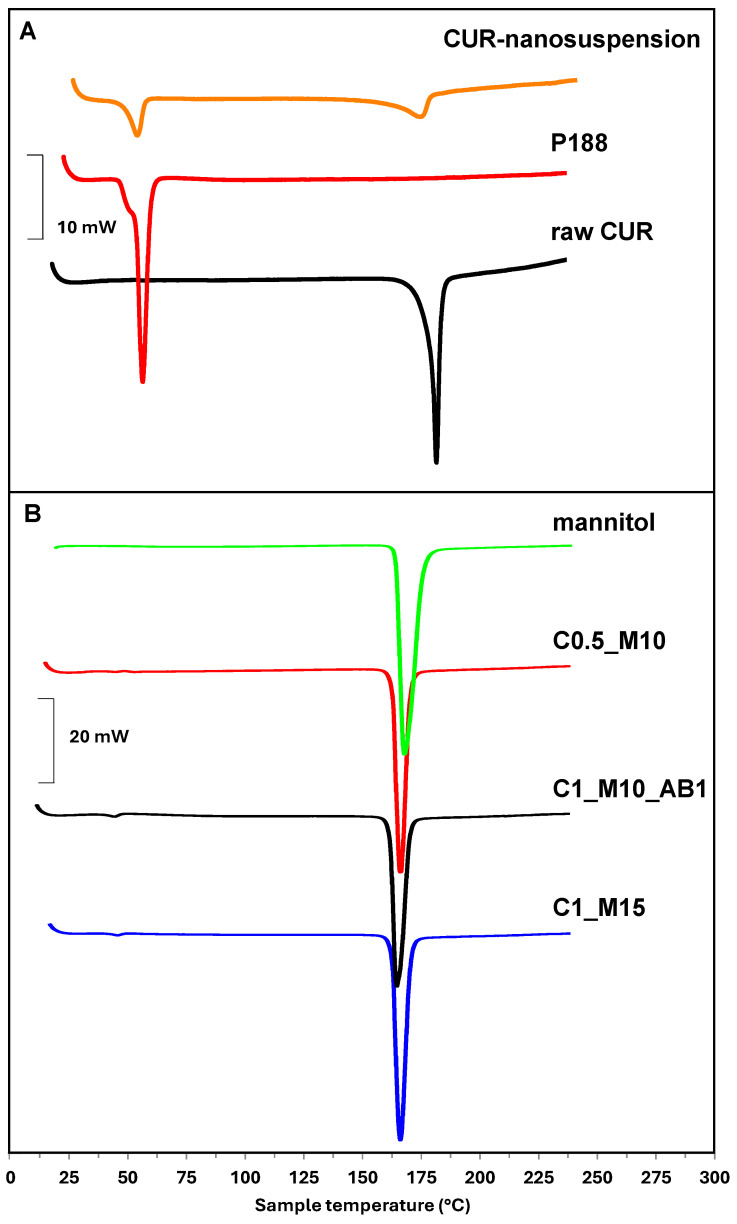
Representative DSC thermograms of (**A**) raw curcumin, P188 and CUR-nanosuspension; (**B**) mannitol, C0.5_M10, C1_M10_AB1 and C1_M15 samples.

In particular, the thermogram of CUR-nanosuspension (Figure 3A) shows two endothermic peaks (at 50.52 °C and 167.40 °C) which are almost superimposable (slightly shifted at lower temperatures) with those recorded on raw P188 (at 56.80 °C) and CUR (179.05 °C) separately. This shift can be attributed to the production process of the nanosuspension. In Panel B, the thermograms of raw mannitol and C0.5_M10, C1_M10_AB1 and C1_M15 samples are reported as representative of all samples. These samples were selected among all, with C0.5_M10 and C1_M15 being the samples with the lowest and highest concentrations of CUR-NC and mannitol, respectively, while C1_M10_AB1 was chosen because it is the only sample produced with AB.

As can be seen, these thermograms show a high endothermic peak at 169.03 °C exactly corresponding to the one observable in the raw mannitol thermogram, while the peaks of CUR and P188 are barely visible due to the predominant presence of the matrix compared to the drug [39]. Only the peak around 50 °C is visible with greater intensity as the CUR concentration in the sample increases, in the order C1_M10_AB1 > C1_M15 > C0.5_M10.

### 2.3. Dissolution and Release Profiles

The microparticle matrix serves as a carrier for proper aerosolization within the bronchial tree. To achieve an optimal dissolution of poorly soluble compounds, a hydrophilic excipient is often preferred as a matrix, as it can attract water upon contact with the biological fluids [40]. Given the high water solubility of mannitol, the microparticle matrix is expected to dissolve when it comes into contact with biological fluids and releases the nanocrystals [41]. This step is essential to achieving the advantages that the nanosized drug offer, like improved penetration across the pulmonary epithelium and increased dissolution velocity [34]. Therefore, a multi-step process is expected, including (i) the dissolution of the mannitol matrix, (ii) the release of the nanocrystals and (iii) their dissolution.

**Figure 4:**
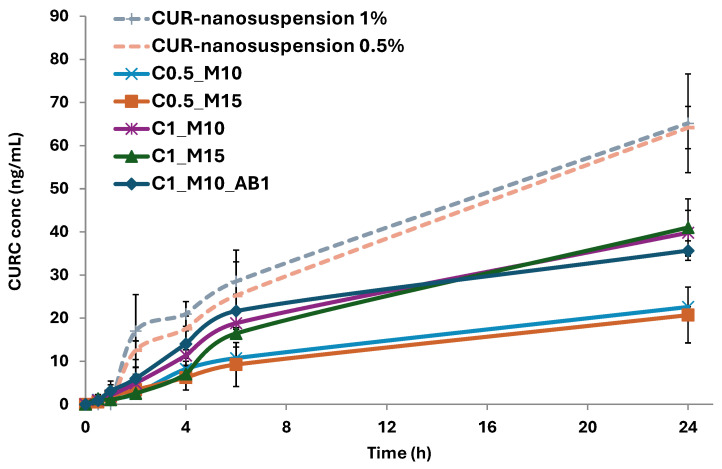
Release/dissolution profiles of pure CUR-nanosuspension and the CUR-NC loaded microparticles in simulated lung fluid (SLF) at 37 °C. (*n* = 3).

To this aim, the dissolution/release profiles of the produced microparticles were studied in simulated lung fluid (SLF) under sink conditions and compared with the dissolution/release of the pure nanocrystals (Figure 4). To allow for a direct comparison, the dissolution/release experiments were carried out using CUR-nanosuspension or microparticle samples containing the same amount of CUR. To better assess whether the formulations release the active ingredient at a comparable rate, the similarity factor (f_2_)—a statistical tool widely used in the context of pharmaceutical release testing—was also calculated (Table 2), where release/dissolution profiles of couples of samples showing an f_2_ ≥ 50 can be considered equivalent.

As can be clearly seen, the release curves of the CUR-nanosuspension in the two samples at different concentrations are statistically similar (f_2_ = 51), highlighting that the release rate is independent of the amount of drug loaded in the dialysis tubes. The two samples showed higher values compared to the microparticles, which is most likely due to the fact that the nanocrystals are directly in contact with the medium, in contrast to the microparticles that need to first dissolve and release its content, thus confirming the hypothesized multi-step release–dissolution process. The microparticles exhibit a gradual release in the incubation medium, without any remarkable burst effect, as already previously observed with mannitol-based microparticles [42]. The first hours of testing are crucial to analyzing the differences among the produced samples. In fact, the C0.5_M10 sample showed a slower release of embedded CUR compared to the ones with 1% (*w*/*v*). It can be hypothesized that in the case of the samples with higher drug loading, there is more of the drug available in the outer surface of the particle, which can be immediately dissolved in the medium. The comparison between the CUR releases from C0.5_M10 and C0.5_M15 resulted in a value of 58, indicating that the amount of mannitol does not statistically affect the cumulative release of the MP loaded with 0.5% (*w*/*v*) of CUR. On the other hand, when the same comparison is performed on the samples with higher CUR loading (1% *w*/*v*; C1_M10 and C1_M15), the f_2_ value reveals that the mannitol concentration influences the cumulative release in these samples. Zhang et al. investigated large porous microparticles with tailored porosity, highlighting that the formed pore and channels might play a key role in the penetration of the release medium into the microparticle, showing a burst release or faster dissolution [43]. In our study, the statistical comparison of the release curve of the sample with the porogen with the corresponding one without (C1_M10 and C1_M10_AB1) showed the equivalence of the two curves (f_2_ = 53), indicating that the presence of AB during the microparticle formation does not affect the dissolution of the matrix.

Focusing on the cumulative release after 24 h, the released CUR from the samples with lower loading was almost twofold lower than that of the ones with higher drug loading (~21 vs. ~40 ng/mL).

### 2.4. Aerodynamic Properties of Microparticles

**Figure 5:**
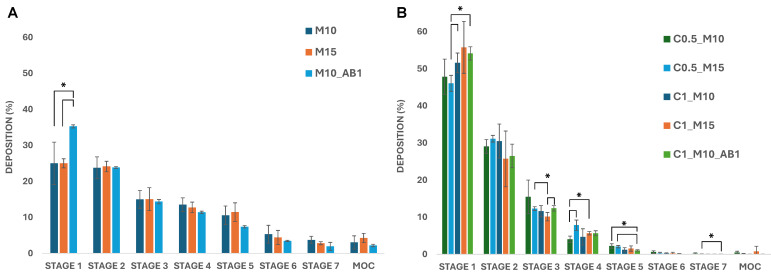
Deposition of (**A**) empty MP and (**B**) CUR-NP MP on the stages of the NGI after testing with Breezhaler^®^ at a flow rate of 90 l min^−1^ (* *p* < 0.05) (MOC = micro-orifice collector).

Once the dissolution properties of the MP were assessed, the samples were also studied to evaluate their aerosolization behavior and the drug deposition in the lungs using a commercial dry powder inhaler (Breezhaler^®^) connected to the NGI (Figure 5), using Tween 80 as a coating agent to prevent particle bouncing. The latter has been described as one of the most promising coating agents for its safe profile and the reproducibility of results [44,45]. Loaded and Empty MP were tested, where RhB was used as a tracking agent in the empty samples.

Regarding Empty-MPs (Figure 5A), M10_AB1 shows a higher deposition in stage 1 compared to the other samples, whereas a comparable deposition pattern with minor variations—but no significant differences—can be observed in the other stages. Interestingly, the incorporation of CUR-NC remarkably affects the deposition of the microparticles compared to the empty ones (Figure 5B). Most of the drug was deposited in the first stages (45–55% in stage 1 and ~30% in stage 2), while lower values were found in the deeper stages.

The deposition patterns were then elaborated to calculate the aerodynamic parameters for the empty formulations (Table 3) and the CUR-NC-loaded ones (Table 4). All of the empty samples reached an ED ≥ 90%, with a respirable fraction ≥ 30% and an average MMAD value of 3.3–4.4. On the other hand, some variations were observed in the loaded samples, with the sample C0.5_M10 being the most promising, showing an ED of approximately 60%, an FPF of 16% and a resulting MMAD of 4.5.

As can be seen from the obtained data, the MMAD is influenced by the presence of aggregates visible in the SEM images, the MMAD values of the samples obtained in the presence of CUR-NC (Table 4) being significantly higher than those of the empty microparticle samples (Table 3). On the other hand, among the samples containing CUR, those obtained using the lowest CUR-NC concentration (0.5% *w/v* in the liquid feed) exhibited a higher FPF value compared to those obtained using the highest concentration (1% *w*/*v*). These results reflect the considerations made earlier based on the visualization of the SEM images and the evaluation of flowability, expressed as H.

Baghdan et al. prepared mannitol microparticles containing polymeric nanoparticles loaded with curcumin that showed a higher FPF%, even though the amount of the drug loaded into the microparticle samples was significantly lower [46]. Nevertheless, the values reported here are in line with previous studies of mannitol-based microparticles incorporating nanocrystals or nanoparticles and suggest an appropriate pulmonary delivery [42,47].

## 3. Materials and Methods

### 3.1. Materials

Curcumin (CUR) and Kolliphor P188 (Poloxamer 188; P188), D-mannitol, ammonium bicarbonate (AB) and rhodamine B (RhB) were purchased from Sigma-Aldrich (Milano, Italy). All solvents and other reagents were of analytical grade.

### 3.2. Preparation of CUR Nanosuspension (CUR-Nanosuspension)

CUR-nanosuspension was produced by a wet ball media milling technique following a previously reported procedure [48]. Briefly, curcumin (1% *w*/*v*) was dispersed in a 0.5% (*w*/*v*) P188 water solution, using an Ultra Turrax basic at 8000 rpm for 6 min. The obtained coarse suspension was then divided into 1.5 mL microtubes with 0.5 g of 0.1–0.2 mm yttrium-stabilized zirconia-silica beads (Silibeads^®^ Typ ZY Sigmund Lindner, Warmensteinach, Germany) and milled for 70 min using a vortex Disruptor Genie^®^ (Scientific Industries, Bohemia, NY, USA) at 3000 vibrations per minute. After the milling process, the CUR-nanosuspension was collected and separated from beads by sieving.

### 3.3. Characterization of CUR-Nanosuspension

The average diameter and polydispersity index (PDI, a measure of the size distribution width) of the CUR-nanosuspension were measured by a Zetasizer nano (Malvern Instrument, Worcestershire, UK) by means of the Dynamic Light Scattering (DLS) technique. A helium-neon laser (633 nm) was used to backscatter the samples at a constant temperature of 25 °C and at an angle of 173°. Using the Zetasizer nano and the M3-PALS (Phase Analysis Light Scattering) method, the zeta potential was also evaluated. The sample was diluted 1:100 before the analysis and each measurement was run in triplicate.

The morphology of the raw CUR powder and the CUR-nanosuspension was examined using an environmental scanning electron microscope (SEM), Zeiss ESEM EVO LS 10 (Oberkochen, Germany), operating at 20 KV in high vacuum mode with a secondary electron detector (SEI). The Agar Automatic Sputter Coater B7341 was used to coat the sample with gold for the raw CUR powder after it had been mounted on an aluminum stub using carbon adhesive discs. Regarding the CUR-nanosuspension, a drop of the sample was first put on a glass slide, allowed to air-dry and then mounted on the stub using the above-mentioned method.

### 3.4. Preparation of the Microparticle Samples

The microparticle samples were prepared by the spray drying (SD) method, using the Mini Spray-Dryer Buchi B290 (Buchi, Milan, Italy). The spray drying process was performed according to the following parameters: inlet T: 110 °C; outlet T: 60–66 °C; aspiration: 100%; feed pump: 10% (3 mL/min); atomizer nozzle: 1.4 µm; used gas: compressed air. The liquids to be nebulized were prepared by adding mannitol to CUR-nanosuspension 1 or 0.5 (*w*/*v*) at a concentration of 10 or 15% (*w*/*v*). When present, the porogen ammonium bicarbonate (AB) was added to the dispersion at a concentration of 1% (*w*/*v*). The control group, consisting of empty mannitol microparticles, was prepared with the addition of Rhodamine B at a concentration equal to 0.05% (*w*/*v*), as a tracing agent for subsequent analyses. At the end of the process, the powder collected from the cyclone in the product vessel was recovered and stored in a dryer, protected from light and moisture. The compositions of all the obtained samples are reported in Table 5.

### 3.5. Evaluation of Bulk and Tapped Density

The syringe method was used to measure the tapped density of all the microparticle samples [37]. To determine the bulk density (ρ_bulk_), each powder was filled into a 1 mL graduated syringe, recording the weight of powder needed. Subsequently, the tapped density (ρ_tapp_) was calculated using the volume measured by tapping the syringe 100 times at a height of 2.5 cm on a level surface, until the volume was constant. Each measurement was performed three times. The Hausner Index (H) was then calculated according to the following equation:

(1) $$H=\frac{\rho tapp}{\rho bulk}\times 100\%$$

### 3.6. Evaluation of Microparticles’ Morphology and Geometric Diameter (d_geom_)

To evaluate the particle morphology, an SEM microscope (Phenom™ ProX Desktop SEM, Thermo Fisher Scientific, Milan, Italy) was used. Each sample was placed on a carbon-coated steel stub, which was sputter-coated with gold to a thickness of about 10 nm and before analysis.

The average geometric diameter (d_geom_) of each sample was calculated by measuring more than 500 particles on SEM images using the ImageJ 1.53k software (National Institutes of Health, Bethesda, MD, USA).

### 3.7. Drug Loading (DL%) Evaluation

The drug loading (DL%), expressed as a weight percentage of the loaded CUR on the microparticle sample, was evaluated by UV-Vis Spectroscopy. In detail, 5 mg of each sample was dissolved in 10 mL of an H_2_O: EtOH mixture (1:1). The solutions were further diluted with ethanol and then analyzed by UV-visible spectroscopy using the multi-plate reader Synergy 4 (BioTek, Winooski, VT, USA) and a wavelength λ = 421 nm. The DL was calculated as:

(2) $$DL\%=\frac{mg\;CUR}{total\;sample\;weight}\times 100$$

### 3.8. Differential Scanning Calorimetry (DSC) Analysis

A DSC 131 Evo instrument (Setaram, Newark, CA, USA) was used to perform the DSC analysis. Before the analysis, the CUR-nanosuspension was frozen at −30 °C and freeze-dried using a Lyovapor L200 (Buchi, Milan, Italy), whereas the microparticle samples were used as spray-dried powders. Mannitol was used as purchased. Each sample was placed inside a sealed aluminum pan and heated/cooled at intervals of 20–240 °C, with a scanning rate of 5 °C min^−1^ for heating and 10 °C min^−1^ for cooling. For each analysis, approximately 8–10 mg of the sample was used.

### 3.9. Dissolution/Release Studies

Dissolution/release studies were carried out for 24 h in Simulated Lung Fluid 4 (SLF, pH 7.4, DPPC 0.2% (*w*/*v*)) [49]. For the microparticles, 5 mg of each sample was dispersed in a dialysis tube, Pur-a-Lyzer (regenerated cellulose membrane, MWCO 3.5 KDa), with 800 μL of SLF. For the CUR-nanosuspension, 15 or 30 uL of the sample—corresponding to the amount of CUR in the C0.5 and C1 microparticles series, respectively—was added in the dialysis tube, and then it was filled with 800 μL of SLF. The tubes were then inserted in a beaker with 80 mL of SLF at 37 °C under magnetic stirring. In total, 10 mL of the medium was withdrawn at set timepoints (0.5, 1, 2, 4, 6, 24 h) from the receiving department and replaced with the same volume of SLF to maintain sink conditions [50]. The withdrawn samples were frozen, freeze-dried and resuspended in 2 mL of methanol for HPLC analysis. The test was run in triplicate for each formulation.

### 3.10. HPLC Analysis

Curcumin was quantified by HPLC using a liquid chromatograph, Alliance 2690 (Waters Corp, Milford, MA, USA), equipped with a multi λ fluorescence detector and a computer integrating apparatus (Empower 3), with a Sunfire C18 column (3.5 µm, 4.6 mm × 150 mm, Waters). A mixture of acetonitrile, water and acetic acid (95:4.84:0.16 *v*/*v*) was used as a mobile phase, eluted at flow rate of 0.5 mL/min. The calibration curve (10–200 ng/μL) was obtained using standard working solutions. Calibration graphs were plotted according to linear regression analysis, which gave a correlation coefficient value (R2) equal to 0.999. Curcumin was determined using a wavelength excitation of λ = 415 nm and an emission of λ = 523 nm.

### 3.11. Aerodynamic Behavior

Aerodynamic parameters were evaluated using a Dry Powder Inhaler (DPI) Breezhaler^®^ and a Next-Generation Impactor (NGI, Apparatus E, Eur. Ph. 10, Copley Scientific Ltd., Nottingham, UK). The NGI was equipped with a pre-separator between the throat and stage 1 to separate large particles, as expected for DPI [51]. All collection plates, including the pre-separator, were coated with a 1% Tween 80 ethanol solution, which was left to dry for 30 min. This is necessary to avoid rebound phenomena of particles and determine the exact quantity of particles that reaches each stage of the NGI [52]. For each experiment, three capsules (size 3) were filled with 10 mg of the formulation. Every capsule was inserted into the DPI, which was connected to the NGI. After having pierced the capsule with the DPI, the test was carried out for 2.7 s, with a flow of 90 L/min, to allow for a passage of air volume of approximately 4 L [53]. After nebulizing the contents of three capsules, the powder deposited in the induction port, the pre-separator and the seven stages and MOC of the NGI was collected using a mixture H_2_O: EtOH (1:1). The solutions obtained were appropriately diluted and analyzed by UV–visible spectroscopy using the multi-plate reader Synergy 4 (BioTek, Winooski, VT, USA). Rhodamine B, used as an agent tracer in the empty formulations, was determined at the wavelength λ = 562 nm, whereas samples containing CUR were analyzed at the wavelength λ = 421 nm. The standard calibration curves were obtained using the standard solutions (50–1 μg/mL for curcumin and 1000–200 μg/mL for rhodamine). The regression analysis linear of the curves obtained from the experimental data showed a value (R^2^) < 0.99.

The amount of the drug deposited in each stage was converted to percent cumulative mass and plotted against the diameter values cut-off of the different stages to calculate the Mass Median Aerodynamic Diameter (MMAD) and Geometric Standard Deviation (GSD), without including the amount of the drug deposited in the induction port, due to the impossibility of defining a precise upper size limit for particles deposited in this section [54]. The MMAD values were extrapolated from the curve obtained according to the indications of the European Pharmacopeia, and GSD was determined accordingly. Furthermore, the emitted dose (ED; drug collected in the mouthpiece, IP, PS, and impaction cups) and the ED% (ratio of the ED to the initial mass of sample tested) were determined by analyzing the data that were obtained. Additionally, the interpolation of the cumulative particle size curve between stages 1 and 2 was used to evaluate the fine particle fraction (FPF%), which is the mass percentage of the aerosol particles with an aerodynamic diameter smaller than 5.0 µm [55,56].

### 3.12. Statistical Analysis

Unless otherwise indicated, the results are presented as the mean ± standard deviation of a minimum of three independent measures. Student’s *t*-test was used to compare two samples, and multiple comparisons of means (one-way ANOVA and post hoc Tukey HSD test) were used to evaluate statistical differences between groups.

A statistically derived mathematical parameter (similarity factor, f_2_) was used to compare the release profiles of the different samples [57]:

(3) $$f_{2}=50\times \log\left\{{\left[1+\frac{1}{n}\sum_{t=1}^{n}{\left(S_{1t}-S_{2t}\right)}^{2}\right]}^{-0.5}\times 100\right\}$$

S_1t_ represents the drug’s released percentage in sample 1, and S_2t_ represents the drug’s released percentage in sample 2 at time *t*. The same time points (*n*) were considered to assess the release profiles, with the first sampling time when drug dissolution was ≥85% being the end point.

The European Medicines Agency and the US Food and Drug Administration both state that when the f2 value falls between 50 and 100, it is verified that the two curves are the same or equivalent [58,59].

## 4. Conclusions

In this study, mannitol microparticles entrapping curcumin nanocrystals were developed as an inhalable powder as a possible treatment for respiratory diseases. First, a CUR nanosuspension was prepared by wet ball media milling starting from raw CUR, leading to the production of nanocrystals with a homogeneous size distribution. The obtained nanosuspension was then used as a vehicle for spray drying, obtaining powders made by CUR nanocrystals dispersed into mannitol microparticles.

The overall analysis showed that the incorporation of CUR nanocrystals into the microparticles affected their morphological properties compared to the empty ones, influencing the geometrical diameter and flowability. A multi-step dissolution and release behavior was observed in the in vitro studies, highlighting the influence of the mannitol concentration on the CUR release.

Additionally, the nebulization studies revealed that the inclusion of CUR nanocrystals affected the aerodynamic parameters, while optimal MMAD values for pulmonary delivery (<5 µm) were still maintained.

## Figures and Tables

**Table 1:** Characteristics of the microparticle samples in terms of geometric diameter (dgeom), tapped density (ρ_tapp_), bulk density (ρ_bulk_), Hausner Index (H), Yield, drug loading (DL%) and entrapment efficiency (EE%).

Samples	d_geom_ (µm)	ρ_tapp_ (g/mL)	ρ_bulk_ (g/mL)	Hausner Index (H)	Yield (% *w*/*w*)	DL (%)	EE (%)
M10	3.2 ± 0.3	0.46 ± 0.01	0.26 ± 0.02	1.77	51.6	-	-
M10_AB1	5.4 ±1.3	0.36 ± 0.01	0.21 ± 0.01	1.71	50.7	-	-
M15	4.8 ± 0.6	0.45 ± 0.04	0.31 ± 0.02	1.45	53.6	-	-
C0.5_M10	2.9 ± 1.2	0.34 ± 0.01	0.26 ± 0.02	1.31	48.4	3.5 ± 0.1	75 ± 2
C0.5_M15	3.2 ± 1.3	0.36 ± 0.02	0.27 ± 0.01	1.33	49.0	2.4 ± 0.2	75 ± 6
C1_M10	3.8 ± 0.4	0.33 ± 0.01	0.21 ± 0.01	1.57	36.7	8.6 ± 0.5	98 ± 5
C1_M15	4.5 ± 0.7	0.35 ± 0.01	0.23 ± 0.01	1.52	41.8	6.0 ± 0.1	100 ± 1
C1_M10_AB1	N.D. ^1^	0.37 ± 0.04	0.21 ± 0.01	1.76	38.1	8.4 ± 0.2	97 ± 2

^1^ N.D. not determined.

**Table 2:** Similarity factor (f_2_) of the CUR release profiles from the different formulations under sink conditions.

*vs*	C0.5_M10	C0.5_M15	C1_M10	C1_M15	C1_M10_AB1	CUR-Nanosuspension 1%	CUR-Nanosuspension 0.5%
C0.5_M10		**58**	20	20	18	1	0
C0.5_M15			17	18	16	0	0
C1_M10				47	**53**	13	10
C1_M15					35	11	9
C1_M10_AB1						13	11
CUR-nanosuspension 1%							**51**
CUR-nanosuspension 0.5%							

**Table 3:** Aerodynamic parameters of the empty mannitol MP: emitted dose (ED%), fine particle fraction (FPF%), mass median aerodynamic diameter (MMAD), geometric standard deviation (GSD) and dispersibility index (DI). Small letters indicate a couple of values of the same measured parameter that are statistically different (*p* < 0.05).

Parameters	M10	M15	M10_AB10
ED%	95.3 ± 3.6	89.0 ± 5.0	94.8 ± 3.8
FPF (%)	35.3 ± 2.2 ^a^	36.6 ± 1.4 ^b^	29 ± 1.2 ^ab^
MMAD (µm)	3.3 ± 0.7	3.3 ± 0.3 ^a^	4.4 ± 0.1 ^a^
GSD	2.7 ± 0.1	2.8 ± 0.4	2.9 ± 0

**Table 4:** Aerodynamic parameters of the CUR-NP-loaded mannitol MP: emitted dose (ED%), fine particle fraction (FPF%), mass median aerodynamic diameter (MMAD), geometric standard deviation (GSD) and dispersibility index (DI). Small letters indicate a couple of values of the same measured parameter that are statistically different (*p* < 0.05).

Parameters	C0.5_M10	C0.5_M15	C1_M10	C1_M15	C1_M10_AB1
ED%	61.4 ± 2.8 ^cf^	49.3 ± 4.3 ^bdfg^	60.8 ± 5.7 ^ab^	40.9 ± 3.9 ^acde^	63.3 ± 0.9 ^eg^
FPF (%)	16.2 ± 1.4 ^ace^	16.5 ± 1.8 ^bdf^	11.7 ± 0.3 ^ab^	11.9 ± 2.2 ^cd^	10.8 ± 0.7 ^ef^
MMAD (µm)	4.5 ± 0.2	4.8 ± 0.1	4.7 ± 0.3	5.2 ± 0.6	4.9 ± 0.1
GSD	2.9 ± 0.3	2.7 ± 0.2 ^a^	3.1 ± 0.3	3.6 ± 1.2	3.3 ± 0.1 ^a^

**Table 5:** Compositions of the prepared mannitol microparticles.

Sample	Composition (% *w*/*v*)		
	Curcumin	Mannitol	Ammonium Bicarbonate
M10	-	10	-
M10_AB1	-	10	1
M15	-	15	-
C0.5_M10	0.5	10	-
C0.5_M15	0.5	15	-
C1_M10	1	10	-
C1_M15	1	15	-
C1_M10_AB1	1	10	1

## Data Availability

The data are available from the authors upon reasonable request.

## Supplementary Materials

The following supporting information can be downloaded at: https://www.mdpi.com/article/10.3390/ph17121708/s1, Supplementary Figure S1. CUR-nanosuspension size distribution of six replicates measured through DLS.
