# Supplementary material for: Encapsulation of Nanocrystals in Mannitol-Based Inhalable Microparticles via Spray-Drying: A Promising Strategy for Lung Delivery of Curcumin
Source: Pharmaceuticals (Basel). 2024 Dec 18;17(12):1708. doi: 10.3390/ph17121708 (PMC11676507; doi:10.3390/ph17121708)
Supplement: Supplementary file 1 [file pharmaceuticals-17-01708-s001.zip › pharmaceuticals-3366353-supplementary.pdf]

pharmaceuticals

MDPI

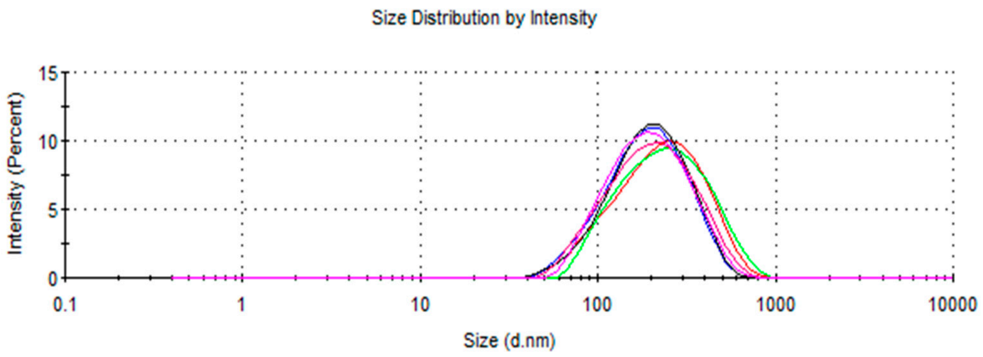

Supplementary Figure S1. CUR-nanosuspension size distribution of six replicates measured through DLS.
